# Diagnosing pancreatic cancer in general practice: a cross-sectional study on associations between suspicion of cancer, urgent referral and time to diagnosis

**DOI:** 10.1080/02813432.2022.2036491

**Published:** 2022-02-11

**Authors:** Line F. Virgilsen, Linda A. Rasmussen, Peter Vedsted, Henry Jensen

**Affiliations:** a Research Centre for Cancer Diagnosis in Primary Care, Research Unit for General Practice, Aarhus, Denmark; b Department of Clinical Medicine, Aarhus University, Aarhus C, Denmark

**Keywords:** Denmark, pancreatic neoplasms, early detection of cancer, general practice, cancer patient pathway

## Abstract

**Objective:**

This study aimed to investigate the first point of contact in patients diagnosed with pancreatic cancer, and to study factors associated with the GP’s suspicion of cancer, Cancer Patient Pathway (CPP) referral and long diagnostic interval.

**Design:**

Cross-sectional study combining register and survey data.

**Patients:**

Patients with incident pancreatic cancer recorded in the Danish National Patient Register (n = 303).

**Main outcome measures:**

The patient’s first point of symptoms presentation, GP’s cancer suspicion, CPP referral and diagnostic interval.

**Results:**

General practice was the first point of contact for 85.5% of the population. At the first consultation, cancer was suspected in 32.7% and 22.9% were referred to a CPP. The GPs were more likely to suspect cancer or serious illness in patients aged >70 years (prevalence rate ratio (PRR) 1.34, 95% CI 1.09–1.66) and among patients with high comorbidity (PRR 1.23, 95% CI 1.04–1.47). A CPP referral was less likely among patients with low education. The median diagnostic interval was 39 days (interquartile range: 15–72). When the GP initially did not suspect cancer, the likelihood of longer diagnostic interval increased.

**Conclusion:**

The majority of patients with pancreatic cancer began their diagnostic route in general practice. Diagnosing pancreatic cancer swiftly in general practice was challenging; the GP did often not initially suspect cancer or refer to a CPP and several of the patient characteristics were associated with the GPs initial suspicion of cancer or CPP referral. Thus, there may be room for improvements in the diagnostics of pancreatic cancer in general practice.

**Key points**
- Patients with pancreatic cancer have a poor prognosis, as pancreatic cancer is often diagnosed in late stage.
- The majority of patients with pancreatic cancer began their diagnostic process in general practice.
- General practitioners (GPs) suspected cancer at the first consultation in one out of three patients with pancreatic cancer; more often in older and comorbid patients.
- The GPs suspicion of cancer was associated with urgent referral and shorter time to diagnosis.

## Background

Danish patients with pancreatic cancer have a poor prognosis with an estimated 5-year survival of less than 5% [1]. One explanation is that the majority of these patients are diagnosed with advanced disease stage and approximately 80% are not candidates for curative surgery [2]. To improve the prognosis in this patient group, more focus is needed on the possibilities to diagnose pancreatic cancer earlier in time [3].

As screening is not currently available for patients with pancreatic cancer [4], the first point of presentation is primarily through emergency presentation, incidental findings at hospitals in connection with investigation of other diseases, or GP referral (e.g. two-week-wait in the UK and Cancer Patient Pathways (CPP) in Denmark) [5–7]. Only few studies exist on the first point of presentation for patients with pancreatic cancer. An English study based on register data reported that 47% of patients were diagnosed after presentation in general practice [5], whereas two European studies based on survey data and interviews found that 70%–75% of the patients presented first in general practice [6,8]. One of these studies found that about half of these patients were referred to the two-week-wait system [8], which tends to reduce the time to diagnosis for most cancer types, including pancreatic cancer [9,10]. However, no data exist from a Nordic healthcare system.

Urgent referral to cancer diagnostics is often guided by symptoms [7]. Yet, pancreatic cancer is typically characterised by vague and unspecific symptoms [4,11,12]. This complicates the patients’ healthcare seeking [8] and the GP’s suspicion of cancer [9], which is a strong indicator for diagnostic action and shorter time to diagnosis in other cancer types [9]. So far, the evidence is sparse on the time intervals in the diagnostic journey for patients with pancreatic cancer [6,8,10,13,14], and little is known about the factors that might provide more rapid diagnosis of pancreatic cancer [6,8].

To obtain a faster and optimal diagnostic process hopefully leading to improvements in the survival of pancreatic cancer patients, more knowledge is needed about the diagnostic pathway. Therefore, the aim of this study was to investigate the first point of contact in patients diagnosed with pancreatic cancer, and to study factors associated with the GP’s suspicion of cancer, CPP referral and long diagnostic interval.

## Material and methods

### Setting

This study was conducted in Denmark, which runs a tax-funded healthcare system with free and equal access for all citizens to most health services. A total of 98% of the population is registered with a general practice who acts as gatekeeper to the secondary health care system, except for emergencies, ear-nose-throat specialists and eye specialists who have direct access [15]. Since 2008/09, CPPs and standardised guidelines have been adopted to ensure equal, prompt and uniform diagnosis and treatment of most cancer types within specific time frames.

### Design, study population and data collection

This study was conducted as a cross-sectional study and based on data from a series of six cohorts among newly diagnosed cancer patients in Denmark collected between 2005 and 2016. For this study, we included patients from the cohort of 2010 and 2016 as these cohorts included data on pancreatic cancer patients and because all Danish Cancer Patient Pathways were implemented from 2010.

We included patients diagnosed with pancreatic cancer (International Classification of Diseases (ICD) Revision 10 code: C25 (except C254, endocrine pancreas) and C241, Ampulla of Vater) with no previous history of cancer. Patients from the two cohorts were identified in the Danish National Patient Register (DNPR) [16] in the period from 1 May to 31 August 2010 or from 1 July to 31 December 2016. The general practice of each included patient was identified in the Danish National Health Service Register [17]. The GP involved in the first presentation of symptoms, or who was most familiar with the patient, was asked to fill in the questionnaire based on their electronic patient record, to ensure that the responses were based on the observations made at the time of the first contact in the cancer course. Questionnaires were sent to the GPs of alive patients on a monthly basis in the data collection period to reduce the time span between the diagnosis and receipt of the questionnaire. However, in 2016, GP participation required written consent from the patient according to the Danish Health Act. For deceased patients in 2016, the Danish Patient Safety Authority granted permission for the GP to be contacted without patient consent so the GPs of these patients received the questionnaire 6–12 months after the patient was diagnosed with cancer. The GP questionnaires were developed by researchers at the Research Unit for General Practice in Aarhus, Denmark and the included time intervals followed the definition from the Aarhus Statement [18]. The questionnaires were pilot tested among GPs prior to data collection where minor changes were made to ensure each question were understood as intended.

### Main variables

All data sources were linked at a personal level through the unique civil registration number assigned to all permanent Danish residents [19].

Four main variables were obtained from the GP questionnaire, and the responses were based on the GPs’ clinical evaluation and information from their medical records. First, the first point of presentation was obtained from the question: “Based on your current knowledge, where did the patient present for the first time before the diagnosis?” Screening is not an option in this patient group, and none of the GPs responded that the patient was diagnosed through specialised private practice or out-of-hours primary care. Thus, the possible routes included: presentation in general practice, diagnosed at the hospital after a contact for other disease, emergency presentation or other route. Second, GP’s suspicion of cancer or serious illness was based on the question: “When the patient consulted general practice for the first time, what was your overall evaluation of the patient?” Three response options were: suspected cancer, suspected serious illness, did not suspect cancer or serious illness. Third, GP referral to a CPP after the first cancer-related presentation had the response options: yes and no. Fourth, the diagnostic interval was defined based on the Aarhus Statement [18] and assessed by calculating the interval between the date of the first presentation of cancer-related symptoms in general practice (as assessed by the GP and recorded in the medical record) and the date of diagnosis [18], which was obtained from the DNPR. The diagnostic interval was defined as the number of days between these two dates. Negative values were coded as 0 days, and intervals longer than one year were coded as 365 days in accordance with the procedures applied by the International Cancer Benchmarking Partnership (ICBP).

In addition, we obtained information on the patient’s health and sociodemographic characteristics. Sex and age at diagnosis were obtained from the Danish Civil Registration System [19]. Statistics Denmark provided data on the patient’s highest attained education, which was categorised according to the International Standard Classification of Education (ISCED) of the United Nations Education, Scientific and Cultural Organization (UNESCO) into short (≤10 years of education), medium (11–15 years of education) and long (>15 years of education), and civil status was dichotomised into living alone or married/cohabiting. Charlson’s Comorbidity Index (CCI) score was calculated from diagnoses (excluding cancer) recorded in the DNPR for up to ten years preceding the pancreatic cancer diagnosis, and CCI scores were categorised into none (score 0), low (scores 1–2) or high (scores >2). Previous GP attendance was measured as the number of face-to-face consultations in general practice in the 12–24 months preceding the cancer diagnosis recorded in the Danish Health Service Register [20], and these were categorised based on the cut-off at the 50th and 75th percentiles, i.e. 0–1, 2–5 and >5 consultations.

### Statistical analyses

The point of first presentation was described by proportions and expressed by percentages. Generalised linear models (GLMs) with log link for the Poisson family were used to study the association between patient characteristics and the likelihood of the GP to suspect cancer or serious illness. GLMs were also used to study patient characteristics, GP suspicion, the likelihood of CPP referral and the likelihood of long diagnostic interval. A long diagnostic interval was defined as the 75th percentile for the total population, i.e. 72 days. To comply with the data protection regulations (i.e. values less than 5 observations at the 75% cut-off are suppressed to prevent disclosure), a pseudo-variable for the diagnostic interval was generated using a method described elsewhere [21]. In all analyses, an unadjusted model was presented and followed by a model adjusted for sex, age, year of diagnosis and GP suspicion of cancer (except for GP suspicion in the analysis in which suspicion was the dependent variable).

Stata software, version 16.0, was used for all statistical analyses.

## Results

In total, 438 patients were eligible for inclusion, and 69.2% (*n* = 303) of the GPs responded to the questionnaire.

### Point of first presentation

The GPs provided information on the point of first presentation for 303 patients, and 259 (85.5%) of these patients presented with initial symptoms in general practice. The remaining 14.5% were diagnosed at the hospital after a contact related to another disease (6.9%), emergency presentation (5.6%) or other reasons (2.0%). Among the patients presenting to general practice, a higher proportion of patients were aged 70–79 years and had no comorbidity compared with patients presenting elsewhere (Table 1).

**Table 1.** Patient characteristics according to GP involvement (*n* = 303).

	GP involved		GP not involved		Total	
	*n*	(% column)	*n*	(% column)	*n*	(%)
Total	259	(100.0)	44	(100.0)	303	(100)
**Age groups (years)**						
<70	**108**	**(41.7)**	**24**	**(54.5)**	132	(43.6)
≥70–79	**102**	**(39.4)**	**8**	**(18.2)**	110	(36.3)
≥80	**49**	**(18.9)**	**12**	**(27.3)**	61	(20.1)
**Sex**						
Female	119	(45.9)	18	(40.9)	137	(45.2)
Male	140	(54.1)	26	(59.1)	166	(54.8)
**Year of diagnosis**						
2010	131	(50.6)	26	(59.1)	157	(51.8)
2016	128	(49.4)	18	(40.9)	146	(48.2)
**Marital status**						
Cohabiting/married	165	(63.7)	28	(63.6)	193	(63.7)
Living alone	94	(36.3)	16	(36.4)	110	(36.3)
**Education**						
Short	113	(43.6)	23	(52.3)	136	(44.9)
Medium and long^a^	146	(56.4)	21	(47.7)	167	(55.1)
**Charlson’s Comorbidity Index**						
None (0)	**159**	**(61.4)**	**18**	**(40.9)**	177	(58.4)
Mild (1-2)	**58**	**(22.4)**	**14**	**(31.8)**	72	(23.8)
High (2 or more)	**42**	**(16.2)**	**12**	**(27.3)**	54	(17.8)
**Previous use of primary care** ^b^						
Infrequent attenders (0–1 visits)	60	(23.2)	8	(18.2)	68	(22.4)
Regular attenders (2–5 visits)	109	(42.1)	16	(36.4)	125	(41.3)
Frequent attenders (>5 visits)	90	(34.7)	20	(45.5)	110	(36.3)
**Tumour stage**						
Local/regional	69	(26.6)	9	(20.5)	78	(25.7)
Distant	119	(45.9)	17	(38.6)	136	(44.9)
Missing	71	(27.4)	18	(40.9)	89	(29.4)
**Death within one year after diagnosis**						
Not death	172	(66.4)	28	(63.6)	200	(66.0)
Death	87	(33.6)	16	(36.4)	103	(34.0)
**GP suspicion at the first presentation**						
Suspected cancer	82	(32.7)	NA	–	82	(32.7)
Suspected serious illness	87	(34.7)	NA	–	87	(34.7)
No suspicion	82	(32.6)	NA	–	82	(32.6)
**Cancer Patient Pathway referral at the first GP presentation**						
No	192	(77.1)	NA	–	192	(77.1)
Yes	57	(22.9)	NA	–	57	(22.9)

Significant differences between groups are shown in bold (Pearson’s chi^2^ square test).

Abbreviations: GP: general practitioner; NA: not applicable.

^a^Medium and long education was combined because one of the columns had <5 observations, thus, cannot be reported according to Statistics Denmark’s data protection regulations.

^b^Based on the number of face-to-face consultations with the GP in the 12–24 months prior to diagnosis.

### GP suspicion of cancer

Among the 259 patients who presented symptoms to their GP, the GP suspected cancer in 32.7%, other serious illness in 34.7%, and neither cancer nor other serious illness in 32.6% (Table 1). Male sex and older age were associated with higher likelihood of GP suspicion. Patients with high comorbidity were also more likely to raise suspicion in their GP compared with patients without comorbidity (PRR 1.23, 95% CI 1.04–1.47) (Table 2).

**Table 2.** GP suspicion of cancer or serious illness at the first cancer-related presentation according to the patient’s characteristics (*n* = 251^a^).

	Prevalence rate ratio of GP suspecting cancer or serious illness						
	GP suspicious		Unadjusted			Adjusted^b^	
	N	% (row)	PRR		95% CI	PRR	95% CI
**Total (*n* = 251, No GP suspicion, *n* = 82)**	169	(67.3)	–		–	–	–
**Sex**							
Female (*n=* 111)	68	(61.2)	1		–	1	–
Male (*n=* 140)	101	(72.1)	1.18		(0.98–1.41)	**1.20**	**(1.00–1.43)**
**Age groups (years)**							
18–69 *(n = 104)*	57	(54.8)	1		–	1	–
70–79 (*n=* 100)	73	(73.0)	**1.33**		**(1.08–1.65)**	**1.34**	**(1.09–1.66)**
>80 (*n=* 47)	39	(83.0)	**1.51**		**(1.22-1.88)**	**1.54**	**(1.24-1.91)**
**Year of diagnosis**							
2010 (*n=* 124)	84	(67.7)	1		–	1	–
2016 (*n=* 127)	85	(66.9)	0.99		(0.83–1.17)	0.98	(0.82–1.15)
**Marital status**							
Cohabiting/married (*n=* 161)	107	(66.5)	1		–	1	–
Not married (*n=* 90)	62	(68.9)	1.04		(0.87–1.24)	1.03	(0.86–1.23)
**Education**							
Short (*n=* 108)	80	(74.1)	1		–	1	–
Medium (*n=* 100)	61	(61.0)	0.82		(0.68–1.00)	0.84	(0.69–1.02)
Long (*n=* 43)	28	(65.1)	0.88		(0.69–1.12)	0.94	(0.73–1.21)
**Charlson’s Comorbidity Index**							
None (score 0) (*n=* 154)	100	(64.9)	1		–	1	–
Mild (score 1–2) (*n=* 56)	34	(60.7)	0.93		(0.73–1.19)	0.91	(0.72–1.15)
High (score 3 or more) (*n=* 41)	35	(85.4)	**1.31**		**(1.11–1.56)**	**1.23**	**(1.04–1.47)**
**Previous use of primary care** ^c^							
Infrequent attenders (0–1 visit) (*n=* 56)	38	(67.9)	1.03		(0.82–1.29)	1.13	(0.89–1.42)
Regular attenders (2–5 visits) (*n=* 108)	71	(65.7)	1		–	1	–
Frequent attenders (>5 visits) (*n=* 87)	60	(69.0)	1.05		(0.86–1.28)	1.02	(0.84–1.23)

GP: general practitioner; PRR: prevalence rate ratio; CI: confidence interval.

Significant results are shown in bold.

^a^8 patients with missing information on GP’s suspicion of cancer at the first cancer-related.

^b^Adjusted for sex, age and year of diagnosis.

^c^Based on number of face-to-face consultations with the GP in the 12–24 months prior to diagnosis and categorised based on the cut-of at the 25 and 75% percentiles.

### CPP referral by the GP

Among the 259 patients who presented symptoms to their GP, the GP referred 22.9% of the patients to a CPP at the first presentation (Table 1). Compared with less educated patients, patients with long education were more likely to be referred to a CPP at the first GP consultation (PRR 2.06, 95% CI 1.09–3.88) (Table 3). When the GP did not suspect cancer at the first consultation, the likelihood of CPP referral at the first presentation was reduced (PRR 0.46, 95% CI 0.25–0.85) (Table 3).

**Table 3.** Cancer Patient Pathway referral after the first cancer-related presentation according to the patient’s characteristics (*n* = 249)^a^.

	Prevalence rate ratio of any Cancer Patient Pathway referral at first presentation							
	Cancer Patient Pathway referral, yes				Unadjusted		Adjusted^b^	
	N		% (row)		PRR	95% CI	PRR	95% CI
**Total (*n* = 249)**	57		(22.9)		**–**	**–**	**–**	–
**Sex**								
Female (*n=* 110)	20		(18.2)		1	–	1	–
Male (*n=* 139)	37		(26.6)		1.46	(0.90–2.38)	1.44	(0.89–2.33)
**Age groups (years)**								
18–69 (*n=* 104)	18		(17.3)		1		1	
70–79 (*n=* 100)	29		(29.0)		1.68	(0.99–2.82)	1.54	(0.91–2.60)
>80 (*n=* 45)	10		(22.2)		1.28	(0.64–2.56)	1.13	(0.55–2.30)
**Year of diagnosis**								
2010 (*n=* 125)	28		(22.4)		1	–	1	–
2016 (*n=* 124)	29		(23.4)		1.04	(0.66–1.65)	1.21	(0.77–1.91)
**Marital status**								
Cohabiting/married (*n=* 158)	37		(23.4)		1	–	1	–
Not married (*n=* 91)	20		(22.0)		0.94	(0.58–1.52)	1.13	(0.71–1.81)
**Education**								
Short (*n=* 109)	18		(16.5)		1	–	1	–
Medium (*n=* 98)	25		(25.5)		1.54	(0.90–2.66)	1.40	(0.79–2.49)
Long (*n=* 42)	14		(33.3)		**2.02**	**(1.11–3.69)**	**2.06**	**(1.09–3.88)**
**Charlson’s Comorbidity Index**								
None (0) (*n=* 151)	36		(23.8)		1	–	1	–
Mild (1–2) (*n=* 57)	14		(24.6)		1.03	(0.60–1.76)	1.05	(0.63–1.73)
High (3 or more) (*n=* 41)	7		(17.1)		0.72	(0.34–1.49)	0.70	(0.33–1.48)
**Previous use of primary care** ^c^								
Infrequent attenders (0–1 visit) (*n=* 56)	15		(26.8)		1.33	(0.75–2.35)	1.35	(0.76–2.41)
Regular attenders (2–5 visits) (*n=* 140)	22		(15.7)		1	–	1	–
Frequent attenders (>5 visits) (*n=* 53)	20		(37.7)		1.18	(0.69–2.02)	1.21	(0.73–2.02)
**GP suspicion at the first presentation**								
Suspected cancer (*n=* 80)	31		(38.7)		1	–	1	–
Suspected serious illness (*n=* 86)	12		(14.0)		**0.36**	**(0.20–0.65)**	**0.37**	**(0.20–0.69)**
No suspicion (*n=* 81)	13		(16.0)		**0.41**	**(0.23–0.73)**	**0.46**	**(0.25–0.85)**

GP: general practitioner; PRR: prevalence rate ratio; CI: confidence interval.

Significant results are shown in bold.

^a^ 10 patients with missing information on GP-initiated Cancer Patient Pathway referral after the first cancer-related presentation.

^b^ Adjusted for sex, age, year of diagnosis and GP’s suspicion.

^c^ Based on face-to-face consultations with the GP in the 12–24 months prior to diagnosis and categorised based on the cut-off at the 25 and 75% percentiles.

### Diagnostic interval

The median diagnostic interval was 39 (Inter quartile interval: 15–72) days (median in 2010: 46 days, median in 2016: 31 days) and 10% had a diagnostic interval longer than 173 days. When the GP did not suspect cancer, an almost three-fold higher likelihood of long diagnostic interval was observed (PRR 2.77, 95% CI 1.40–5.49) (Table 4). Patients who were referred to a CPP at the first GP consultation had a non-significant lower likelihood of long diagnostic interval (PRR 0.59, 95% CI 0.28–1.24). The patient’s health and sociodemographic characteristics were not associated with the diagnostic interval (Table 4).

**Table 4.** Long diagnostic interval according to the patient’s characteristics (*n* = 223^a^).

	Prevalence rate ratio of long diagnostic interval (≥72 days)						
	Long diagnostic interval (>72 days)			Unadjusted		**Adjusted** ^b^	
	N		% (row)	PRR	95% CI	PRR	95% CI
**Total (*n* = 223)**	56		(25.1)	**–**	**–**	**–**	–
**Sex**							
Female (*n=* 102)	29		(26.7)	1	–	1	–
Male (*n=* 121)	27		(23.8)	0.87	(0.55–1.37)	0.97	(0.62–1.51)
**Age groups (years)**							
18–69 (*n=* 92)	25		(27.4)	1	–	1	–
70–79 (*n=* 93)	22		(22.6)	0.84	(0.51–1.39)	0.93	(0.55–1.57)
>80 (*n=* 38)	9		(25.6)	0.94	(0.50–1.78)	1.17	(0.63–2.20)
**Year of diagnosis**							
2010 (*n=* 119)	30		(25.2)	1	–	1	–
2016 (*n=* 104)	26		(25.0)	1.01	(0.64–1.59)	0.97	(0.61–1.53)
**Marital status**							
Cohabiting/married (*n=* 144)	34		(23.6)	1	–	1	–
Not married (*n=* 79)	22		(27.8)	1.18	(0.74–1.87)	1.10	(0.69–1.76)
**Education**							
Short (*n=* 100)	27		(26.3)	1	–	1	–
Medium (*n=* 87)	18		(20.7)	0.79	(0.46–1.34)	0.80	(0.47–1.38)
Long (*n=* 36)	11		(32.4)	1.23	(0.70–2.19)	1.32	(0.76–2.31)
**Charlson’s Comorbidity Index**							
None (score 0) (*n=* 137)	35		(26.3)	1	–	1	–
Mild (score 1–2) (*n=* 50)	14		(25.5)	0.96	(0.56–1.67)	0.90	(0.51–1.59)
High (score 3 or more) (*n=* 36)	7		(20.0)	0.73	(0.36–1.51)	0.80	(0.39–1.63)
**Previous use of primary care** ^c^							
Infrequent attenders (0–1 visit) (*n=* 54)	15		(26.3)	1.36	(0.75–2.45)	1.37	(0.74–2.53)
Regular attenders (2–5 visits) (*n=* 94)	21		(21.3)	1	–	1	–
Frequent attenders (>5 visits) (*n=* 75)	20		(27.6)	1.42	(0.83–2.42)	1.40	(0.84–2.33)
**GP suspicion at the first presentation**							
Suspected cancer (*n=* 73)	10		(13.7)	1	–	1	–
*Suspected serious illness* (*n=* 74)	17		(23.0)	1.75	(0.87–3.54)	1.75	(0.84–3.65)
No suspicion (*n=* 72)	27		(37.5)	**2.74**	**(1.43–5.24)**	**2.77**	**(1.40–5.49)**
**Cancer Patient Pathway referral at the first GP presentation**							
No (*n=* 166)	47		(28.3)	1	–	1	–
Yes (*n=* 50)	7		(14.0)	0.49	(0.24–1.03)	0.59	(0.28–1.24)

GP: general practitioner; PRR: prevalence rate ratio; CI: confidence interval.

Significant results are shown in bold.

^a^36 patients with missing information on one of the dates used to calculate the diagnostic interval.

^b^Adjusted for sex, age, year of diagnosis and GP’s suspicion.

^c^Based on number of face-to-face consultations with the GP in the 12–24 months prior to diagnosis and categorised based on the cut-off at the 25 and 75% percentiles.

## Discussion

### Principal findings

This population-based study found that the majority of patients with pancreatic cancer began their route to diagnosis in general practice. In one out of three patients, neither cancer nor serious illness was suspected when the patient first presented in general practice. GPs were more likely to suspect cancer in males, older patients and patients with high comorbidity. One in four patients were referred to a CPP after the first presentation in general practice, and CPP referral was more likely among highly educated patients. GP suspicion was crucial for both CPP referral at the first presentation and shorter diagnostic interval.

### Strengths and limitations

Patients were identified through the population-based DNPR [22]. High data completeness ensured nearly complete identification and minimised selection bias. Questionnaire data was the primary source of data, and prior to data collection, the questionnaires were pilot tested among GPs to enhance the face and construct validity. The questionnaire focused on the first presentation in general practice and not the entire diagnostic process in general practice, which could have provided additional insights for patients with several GP consultations before referral as often is the case with patients diagnosed with pancreatic cancer [23]. The response rate of 70% might have caused selection bias. However, the sub-analyses showed that patients’ health and sociodemographic characteristics were equally distributed among responding and non-responding GPs, yet, a higher proportion of non-respondents (47%) were deceased within one year of the diagnosis compared to respondents (34%) (Data not shown).

Information bias caused by GP recall problems cannot be rejected. Yet, as GPs in Denmark have electronic medical records, which they were encouraged to consult, information bias from this source was minimised. The study included two cohorts of cancer patients diagnosed in 2010 and 2016. We have no systematically collected information on public campaigns in the time between the two cohorts, however, the data relied on GP’s assessment which may be less susceptible to public campaigns. Further, all current CPPs were implemented from 2010 and the proportion of CPP referral and GP suspicion did not differ statistically significantly in the two cohorts (data not shown) indicating no major changes in GP behaviour over time.

Data on symptoms experienced by the patients at the first presentation could have provided additional insights into the mechanisms as symptoms may be linked with the point of first presentation, the GP’s suspicion and the choice of action [9]. Unfortunately, these data was not available in this study as first presented symptoms were not collected in the GP questionnaire.

Due to the relatively small population size, the statistical precision was reduced, and type II errors cannot be ruled out. With a larger population, some of the findings may have reached statistical significance, e.g. the association between previous GP attendance and CPP referral. Finally, the implications for early diagnosis of pancreatic cancer apply only to the patients who presented in general practice and first time cancer patients, as patients with previous cancer were excluded to avoid that the GP suspicion, CPP referral and diagnostic interval was modified by previous experiences.

### Comparison with other studies

For the majority of the included patients, the first point of symptom presentation was in the GP setting. This finding is comparable with the findings in two European studies [6,8] which reported that the majority of patients initially presented in general practice, although the proportion of patients presenting as emergency was larger in both the UK [8] and France [6] than in our study.

The GP suspected cancer in one in three patients at the first presentation, which is lower than reported across all cancer types in Denmark, where the GP suspected cancer at the first presentation in 48% [9]. Still, it is roughly similar to the 27% reported in patients with lung cancer in Denmark [24]. This may reflect that pancreatic cancer often presents with unspecific and vague symptoms, supporting pancreatic cancer as a so-called ‘hard to diagnose’ type of cancer like lung cancer [23].

To our knowledge, the link between a patient’s health and sociodemographic characteristics and the GPs’ suspicion of cancer at the first presentation in general practice has not been studied previously among patients with pancreatic cancer. This study provides new knowledge, which indicates a higher propensity among GPs to suspect cancer or serious illness in older patients, in patients with high comorbidity and, to some extent, in males. In line with previous studies on other cancer types, we found that the GP’s suspicion of cancer was strongly associated with a higher propensity of referring to a CPP after the patient’s first clinical presentation and with shorter diagnostic interval [9,25]. This underlines that the GP’s initial actions are important in healthcare systems with gatekeeping.

Initial CPP referral was observed in 22%, which was comparable to a previous study reporting that 27% of patients with lung cancer were referred to a CPP after the first GP presentation [24]. However, Walter et al. [8] reported that half of the patients with pancreatic cancer presenting to their GP in England were referred to a fast-track pathway. Walter et al. analysed the final referral mode and not the action by the GP at the first presentation. Thus, the different findings in the two studies could indicate that an interval may exist from the first presentation until urgent referral (e.g. CPP) for half of those referred to a fast-track pathway for pancreatic cancer [8].

An interesting finding in this study was that patients with low education were less likely to get a CPP referral at the first consultation, which is somewhat supported elsewhere [25]. Clinical bias has been raised as a potential explanation, as GPs may more actively refer affluent patients [26], or affluent patients may be more successful in articulating and navigating the referral process in the cancer pathway [27]. However, the explanation for this finding remains unclear, and more research should be undertaken.

The diagnostic interval decreased from 2010 to 2016, which is in line with results in cancer patients in general [10,28], although the median diagnostic interval in this study was seven days longer than the diagnostic interval found for all cancer types combined in 2010 [28]. Nevertheless, the median length of the diagnostic interval of 39 days is similar to the diagnostic interval in English patients with pancreatic cancer [8], and it corresponds well with research indicating that patients with pancreatic cancer more often than patients with other types of cancer present three time or more in general practice before referral [23]. The patient’s sociodemographic characteristics were not associated with the diagnostic interval, which could be due to the adjustment of cancer suspicion in the model, which seemed to be the factor with the largest impact on both CPP referral and diagnostic interval. Likewise, another study on pancreatic cancer patients found no association between sociodemographic characteristics and time intervals [8].

### Implications

In countries with GP gatekeeping, the GP plays a crucial role in diagnosing cancer early. This study shows that additional support for the GPs is needed to ensure timely diagnosis of pancreatic cancer. Although a CPP for pancreatic cancer has been implemented in Denmark, three out of four patients with pancreatic cancers were not referred to a CPP at their first cancer-related consultation in general practice. As pancreatic cancer is difficult to diagnose due to vague and unspecific symptoms [2], studies into increased access to diagnostic tools, such as ultrasound, CT and endoscopy, is needed. Further, continuing medical education of GPs is crucial to ensure upgrading of the skills required for diagnosing pancreatic cancer. In light of the results, medical education could draw attention to how the educational level of the patient affect the GPs diagnostic behaviour as this study indicated lower propensity in referral of low educated patients.

## Conclusion

The vast majority of patients with pancreatic cancer began their diagnostic route by presenting symptoms to their GP. The time to diagnosis was prolonged when the GP did not suspect pancreatic cancer. Suspicion of cancer or other serious illness was associated with male gender, older age and high comorbidity. Finally, patients with low education were less often referred to a CPP.
